# A *TNFSF13B* functional variant is not involved in systemic sclerosis and giant cell arteritis susceptibility

**DOI:** 10.1371/journal.pone.0209343

**Published:** 2018-12-26

**Authors:** David González-Serna, Elio G. Carmona, Norberto Ortego-Centeno, Carmen P. Simeón, Roser Solans, José Hernández-Rodríguez, Carlos Tolosa, Santos Castañeda, Javier Narváez, Ferran Martinez-Valle, European GCA Consortium, European Scleroderma Group, Torsten Witte, Thomas Neumann, Julia Holle, Lorenzo Beretta, Luigi Boiardi, Giacomo Emmi, Marco A. Cimmino, Augusto Vaglio, Ariane L. Herrick, Christopher P. Denton, Carlo Salvarani, María C. Cid, Ann W. Morgan, Carmen Fonseca, Miguel A. González-Gay, Javier Martín, Ana Márquez

**Affiliations:** 1 Instituto de Parasitología y Biomedicina "López-Neyra", CSIC, PTS Granada, Granada, Spain; 2 Systemic Autoimmune Diseases Unit, Hospital Clínico San Cecilio, Granada, Spain; 3 Autoimmune Systemic Diseases Unit, Department of Internal Medicine, Hospital Vall d’Hebron, Autonomous University of Barcelona, Barcelona, Spain; 4 Vasculitis Research Unit, Department of Autoimmune Diseases, Hospital Clínic, University of Barcelona, Institut d’Investigacions Biomèdiques August Pi i Sunyer (IDIBAPS), Barcelona, Spain; 5 Department of Internal Medicine, Hospital Parc Tauli, Sabadell, Spain; 6 Department of Rheumatology, Hospital de la Princesa, IIS-Princesa, Madrid, Spain; 7 Department of Rheumatology, Hospital Universitari de Bellvitge, Barcelona, Spain; 8 Hannover Medical School, Hannover, Germany; 9 Klinik für Innere Medizin III, University-Hospital Jena, Jena, Germany; 10 Vasculitis Clinic, Klinikum Bad Bramstedt & University Hospital of Schleswig Holstein, Bad Bramstedt, Germany; 11 Referral Center for Systemic Autoimmune Diseases, Fondazione IRCCS Ca’ Granda Ospedale Maggiore Policlinico di Milano, Milan, Italy; 12 Rheumatology Unit, Department of Internal Medicine, Azienda Ospedaliera Arcispedale Santa Maria Nuova, Istituto di Ricovero e Cura a Carattere Scientifico, Reggio Emilia, Italy; 13 Department of Experimental and Clinical Medicine, University of Florence, Florence, Italy; 14 Research Laboratory and Academic Division of Clinical Rheumatology, Department of Internal Medicine, University of Genova, Genova, Italy; 15 Department of Biomedical Experimental and Clinical Sciences "Mario Serio", University of Firenze, Firenze, Italy; 16 Nephrology and Dialysis Unit, Meyer Children's University Hospital, Firenze, Italy; 17 Centre for Musculoskeletal Research and NIHR Manchester Musculoskeletal Biomedical Research Unit, The University of Manchester, Salford Royal NHS Foundation Trust, Manchester Academic Health Science Centre, Manchester, UK; 18 Centre for Rheumatology, Royal Free and University College Medical School, London, UK; 19 Azienda USL-IRCCS di Reggio Emilia and Università di Modena e Reggio Emilia, Reggio Emilia, Italy; 20 School of Medicine, University of Leeds and NIHR-Leeds Musculoskeletal Biomedical Research Unit, Leeds Teaching Hospitals NHS Trust, Leeds, United Kingdom; 21 Department of Rheumatology, Hospital Universitario Marqués de Valdecilla, IDIVAL, University of Cantabria, Santander, Spain; 22 Systemic Autoimmune Disease Unit, Hospital Clínico San Cecilio, Instituto de Investigación Biosanitaria de Granada (ibs.GRANADA), Granada, Spain; Peking University First Hospital, CHINA

## Abstract

**Background:**

The *TNFSF13B* (TNF superfamily member 13b) gene encodes BAFF, a cytokine with a crucial role in the differentiation and activation of B cells. An insertion-deletion variant (GCTGT→A) of this gene, leading to increased levels of BAFF, has been recently implicated in the genetic predisposition to several autoimmune diseases, including multiple sclerosis, systemic lupus erythematosus, and rheumatoid arthritis. Based on the elevated levels of this cytokine found in patients with giant cell arteritis (GCA) and systemic sclerosis (SSc), we aimed to assess whether this functional variant also represents a novel genetic risk factor for these two disorders.

**Methods:**

A total of 1,728 biopsy-proven GCA patients from 4 European cohorts, 4,584 SSc patients from 3 European cohorts and 5,160 ethnically-matched healthy controls were included in the study. The single nucleotide polymorphism (SNP) rs374039502, which colocalizes with the genetic variant previously implicated in autoimmunity, was genotyped using a custom TaqMan assay. First, association analysis was conducted in each independent cohort using χ2 test in Plink (v1.9). Subsequently, different case/control sets were meta-analyzed by the inverse variance method.

**Results:**

No statistically significant differences were found when allele distributions were compared between cases and controls for any of the analyzed cohorts. Similarly, combined analysis of the different sets evidenced a lack of association of the rs374039502 variant with GCA (P = 0.421; OR (95% CI) = 0.92 (0.75–1.13)) and SSc (P = 0.500; OR (95% CI) = 1.05 (0.91–1.22)). The stratified analysis considering the main clinical subphenotypes of these diseases yielded similar negative results.

**Conclusion:**

Our data suggest that the *TNFSF13B* functional variant does not contribute to the genetic network underlying GCA and SSc.

## Introduction

Autoimmune diseases are complex disorders caused for the combined effect of both environmental and polygenic risk factors. In recent years, genetic studies have identified hundreds of *loci* implicated in the susceptibility of immune-mediated conditions, many of which are shared by different diseases, thus highlighting the existence of shared pathogenic mechanisms in autoimmunity [1].

In this regard, a genetic variant of the *TNFSF13B* (TNF superfamily member 13b) gene has been recently involved in the susceptibility to several autoimmune disorders, including multiple sclerosis (MS), rheumatoid arthritis (RA), and systemic lupus erythematosus (SLE) [2]. *TNFSF13B* encodes BAFF (B-cell activating factor), a cytokine belonging to the tumor necrosis factor (TNF) ligand family with a key role in the differentiation and activation of B cells [3]. The *TNFSF13B* autoimmune-associated variant is an insertion-deletion (GCTGT→A) that creates a shorter 3′ UTR transcript lacking a miRNA binding site. This leads to higher levels of soluble BAFF, which results in an increased number of B cells and a reduction of the circulating monocytes [2]. Interestingly, recent studies have described increased levels of BAFF in giant cell arteritis (GCA) [4, 5] and systemic sclerosis (SSc) patients [6]. Specifically, in GCA, serum BAFF levels appeared to be positively correlated with disease activity and inversely correlated with circulating B cell number [4, 5]. Regarding SSc, it has been demonstrated that patients showing IFN type I signature had higher monocyte BAFF mRNA levels [7]. In addition, BAFF has also been implicated in the production of both IgG and IL6 by B cells [8] and collagen by dermal fibroblasts in SSc patients [9], thus contributing to the inflammatory and fibrotic processes occurring in this disorder.

Taking this into account, we decided to investigate for first time the possible implication of the *TNFSF13B* functional variant in GCA and SSc by analyzing the largest cohorts of patients with these two diseases so far.

## Materials and methods

### Study population

A total of 1,728 biopsy-proven GCA patients, 4,584 SSc patients, and 5,160 ethnically-matched healthy controls, all of them of European origin, were included in the study. Additional information about the case/control sets included in the analysis is provided in Table 1.

**Table 1:** Case/control sets included in the present study.

	GCA			SSc			Controls		
	N	Female (%)	Age^a^ (mean ± SD)	N	Female (%)	Age^a^ (mean ± SD)	N	Female (%)	Age^a^ (mean ± SD)
Spain	891	63.0	83.62 ± 8.71	2,086	89.5	61.76 ± 15.10^b^	3,200	68.1	45.15 ± 11.75
Italy	326	80.2	79.04 ± 7.09^b^	1,105	90.6	58.64 ± 15.53	1,118	44.8^b^	51.12 ± 14.53^b^
Germany	186	78.9	67.53 ± 8.84	-	-	-	470	56.0^b^	55.32 ± 6.96
UK	325	69.2	72.90 ± 7.36	1,393	85.2	63.97 ± 14.39^b^	372	44.5	43.57 ± 8.23^b^
**Total**	**1,728**			**4,584**			**5,160**		

^a^ Age at the sample collection

^b^ Data available for less than 60% of individuals.

Approval from the Comité de Bioética del Consejo Superior de Investigaciones Científicas and the local ethical committees of the different participating centers (Hospital Vall d'Hebron, Hospital Clinic, Hospital Universitario de Bellvitge, Hospital del Mar, Hospital General de Granollers, Hospital de la Santa Creu i Sant Pau, Hospital Universitari Arnau de Vilanova, Consorci Corporació Sanitària Parc Taulí de Sabadell, Hospital Universitario MútuaTerrassa, Hospital Universitario A Coruña, Hosptal Xeral-Calde, Complejo Hospitalario Universitario de Vigo, Hospital Álvaro Cunqueiro, Hospital Carlos Haya, Hospital Virgen de la Victoria, Hospital Regional de Málaga, Hospital Reina Sofía, Hospital Clínico Universitario San Cecilio, Hospital Vírgen de las Nieves, Hospital Vírgen del Rocío, Hospital 12 de octubre, Hospital Clínico San Carlos, Hospital Gregorio Marañón, Hospital La Paz, Hospital La Princesa, Hospital Ramón y Cajal, Hospital Universitario HM Sanchinarro, Hospital Puerta de Hierro, Hospital de Cruces, Hospital Universitario de Galdakao, Hospital Virgen del Camino, Hospital Central de Asturias, Hospital Marqués de Valdecilla, Hospital Universitario Doctor Peset, Hospital La Fé, Hospital Clínico Universitario Lozano Blesa, Hospital Universitario Miguel Servet, Hospital de Son Llàtzer, Hospital Universitario de Canarias, ASST Degli Spedali Civili Di Brescia, Fondazione IRCCS Ca’ Granda Ospedale Maggioire Policlinico di Milano, Università degli Studi di Verona, Ospedale Torrette di Ancona, Azienda Ospedaliera ASMN, University Hospital of Parma, University of Genova, Catholic University of Sacred Heart, Ospedali Riuniti, Università Vita-Salute San Raffaele, University of Firenze, Robert-Bosch-Hospital, Hannover Medical School, University of Luebeck, University of Jena, Leeds Teaching Hospitals NHS Trust, Southend University Hospital NHS Foundation Trust, Ipswich Hospital NHS Trust, King’s College London, University of Glasgow, Newcastle University, Royal Free and University College Medical School, University of Manchester) and informed written consent from all participants were obtained in accordance with the tenets of the Declaration of Helsinki.

GCA patients had a positive temporal artery biopsy (disruption of the internal elastic laminae with infiltration of mononuclear cells into the arterial wall with or without multinucleated giant cells) and fulfilled the 1990 American College of Rheumatology (ACR) classification criteria [10]. SSc patients fulfilled the 1980 ACR preliminary classification criteria for the disease [11] or exhibited at least 3 of 5 features of CREST syndrome (calcinosis, Raynaud’s phenomenon, esophageal dysmotility, sclerodactyly, telangiectasias).

Patients with GCA were stratified according to the presence/absence of the main clinical phenotypes of the disease, polymyalgia rheumatica, jaw claudication, and visual manifestations (transient visual loss including amaurosis fugax, permanent visual loss, or diplopia). SSc patients were stratified according to the extent of skin involvement and autoantibody status as limited cutaneous SSc (lcSSc), diffuse cutaneous SSc (dcSSc), positive for anticentromere antibodies (ACA), and positive for antitopoisomerase antibodies (ATA).

### Genotyping methods

Genomic DNA was extracted from peripheral white blood cells using standard procedures. All individuals were genotyped for the single nucleotide polymorphism (SNP) rs374039502 (1099T>A), which colocalizes with the insertion/deletion variant previously implicated in autoimmunity, using a TaqMan allelic discrimination custom assay (ID: AH0JGPG) with the following primers: forward 5’-*GACAGCATCCCGGTTTTCATTTTAT*-3’ and reverse 5’-*TGTAAACTGTTAAATGAAGTAAACAGTTAAAACTGA*-3’. Genotyping was performed in a 7900HT Fast Real-time polymerase chain reaction system (Applied Biosystems, Foster City, CA, USA).

### Statistical analysis

The overall statistical power of the analysis was calculated using CaTS (http://www.sph.umich.edu/csg/abecasis/CaTS/). Hardy–Weinberg equilibrium (HWE) was tested at a significance level of 0.05. The statistical analysis to compare allelic distributions was carried out using χ^2^ test in Plink (v1.9) (https://www.cog-genomics.org/plink2). Then, results from the different cohorts were combined using inverse variance weighted meta-analysis under a fixed-effects model. Heterogeneity of the ORs across cohorts was assessed using Cochran’s Q test. Odds ratios (OR) and 95% confidence intervals (CI) were obtained according to Woolf’s method. P-values <0.05 were considered statistically significant.

## Results

Genotyping success rate was higher than 95%. Genotypic frequencies did not deviate from HWE.

Results of the association analysis of each individual case/control set and the combined analysis are shown in Tables 2 and 3. First, allele frequencies were compared between cases and controls for each independent cohort. Regarding GCA, a trend for association was observed in the German population (186 cases and 470 controls; p = 0.069); however, no significant association was found for any of the remaining case/control sets, despite of the higher sample size and allele frequency of both the Spanish (891 cases and 3,200 controls) and Italian (326 cases and 1,118 controls) cohorts (Table 2).

**Table 2:** Association analysis of the *TNFSF13B* rs374039502 variant in four independent GCA cohorts and meta-analysis.

		Genotype, N (%)				Allele test	
Cohort	Subgroup (N)	TT	TA	AA	MAF	P-value	OR [95% CI] *
Spain	Controls (n = 3,200)	10 (0.31)	249 (7.78)	2941 (91.91)	4.20	0.170	0.82 [0.62–1.09]
	GCA (n = 891)	0 (0)	62 (6.96)	829 (93.04)	3.48		
Italy	Controls (n = 1,118)	3 (0.27)	125 (11.18)	990 (88.55)	5.86	0.533	0.88 [0.59–1.30]
	GCA (n = 326)	0 (0)	34 (10.43)	292 (89.57)	5.21		
Germany	Controls (n = 470)	0 (0)	19 (4.04)	451 (95.96)	2.02	0.069	1.89 [0.94–3.82]
	GCA (n = 186)	0 (0)	14 (7.53)	172 (92.47)	3.76		
UK	Controls (n = 372)	0 (0)	17 (4.57)	355 (95.43)	2.28	0.977	1.01 [0.50–2.00]
	GCA (n = 325)	0 (0)	15 (4.62)	310 (95.38)	2.31		
Overall meta-analysis	Controls (n = 5,160)					0.421	0.92 [0.75–1.13]
	GCA (n = 1,728)						

GCA, giant cell arteritis; MAF, minor allele frequency; OR, odds ratio; CI, confidence interval.

* OR for the minor allele (A).

**Table 3:** Association analysis of the *TNFSF13B* rs374039502 variant in three independent SSc cohorts and meta-analysis.

		Genotype, N (%)				Allele test	
Cohort	Subgroup (N)	TT	TA	AA	MAF	P-value	OR [95% CI]*
Spain	Controls (n = 3,200)	10 (0.31)	249 (7.78)	2941 (91.91)	4.20	0.326	1.10 [0.91–1.33]
	SSc (n = 2,086)	5 (0.24)	182 (8.72)	1899 (91.04)	4.60		
Italy	Controls (n = 1,118)	3 (0.27)	125 (11.18)	990 (88.55)	5.86	0.822	0.97 [0.75–1.25]
	SSc (n = 1,105)	5 (0.45)	116 (10.50)	984 (89.05)	5.70		
UK	Controls (n = 372)	0 (0)	17 (4.57)	355 (95.43)	2.28	0.893	1.04 [0.60–1.78]
	SSc (n = 1,393)	1 (0.07)	64 (4.59)	1328 (95.33)	2.37		
Overall meta-analysis	Controls (n = 4,690)					0.500	1.05 [0.91–1.22]
	SSc (n = 4,584)						

SSc, systemic sclerosis; MAF, minor allele frequency; OR, odds ratio; CI, confidence interval.

* OR for the minor allele (A).

On the other hand, no association of this polymorphism with SSc was observed in any of the three analyzed cohorts (Table 3).

Accordingly, when the different case/control sets were combined in a meta-analysis, a lack of association of the *TNFSF13B* variant with both diseases, GCA (P = 0.421, OR = 0.92) and SSc (P = 0.936, OR = 1.00), was evident (Tables 2 and 3).

Subsequently, to examine whether this polymorphism might affect specific clinical subgroups, GCA and SSc patients were stratified according to the main clinical manifestations of each disease. Again, the subphenotype analysis yielded similar negative results in each case/control set (data not shown) as well as in the meta-analysis of the different cohorts (S1 and S2 Tables).

## Discussion

Different lines of evidence suggest that BAFF plays a relevant role in the pathogenic process occurring in autoimmune disorders. Interestingly, Belimumab, a monoclonal antibody against BAFF, was recently approved for SLE treatment and is currently undergoing Phase III clinical trial in antineutrophil cytoplasmic antibody (ANCA)-associated vasculitis. Genetic studies also support the role of this cytokine in SLE, MS and RA. In this regard, a *TNFSF13B* insertion-deletion variant, which leads to higher levels of BAFF, has been proposed as a common genetic risk factor in autoimmunity. Elevated levels of BAFF have been found in GCA and SSc patients, thus providing a strong rationale for the study of the *TNFSF13B* functional variant in the genetic predisposition to these disorders.

Large-scale genetic analyses have already been performed in both GCA [12] and SSc [13]; however, since the genotyping platforms used in this kind of studies only include SNPs and small indels, the potential role of other type of genetic variants, which could help to clarify part of the missing heritability of these conditions, remains unexplored. Taking this into account, we decided to investigate the possible implication of the *TNFSF13B* functional variant in the GCA and SSc pathogenesis through a candidate gene approach. However, our data evidenced a lack of association of this genetic variant with both disorders.

Despite the low prevalence of SSc and the low frequency of the analyzed polymorphism, our study had enough statistical power to detect a similar effect to that reported for other autoimmune diseases (~ 80% to detect ORs previously described for MS (1.27), RA (1.24), or SLE (1.44)), therefore, it is unlikely that the lack of association observed herein was due to a type II error. With respect to GCA, although our study had enough statistical power to detect moderate effects (80% to detect an OR≥1.36), a weaker effect of this variant in GCA can not be completely discarded. Nevertheless, according to the results of the meta-analysis of the different GCA cohorts, the minor allele (A) was found to have an opposite effect in this condition (OR = 0.92) compared to that previously described for other diseases. Therefore, it is unlikely that replication studies including larger sample sets result in a different outcome.

Although our data indicated that the *TNFSF13B* deletion is not involved in GCA and SSc, an implication of other polymorphisms within this locus, in low linkage disequilibrium with the variant analyzed here, can not be ruled out. However, it should be noted that no signals within this region were detected in previous large-scale genetic analyses. Another possibility is that genetic variants located in regulatory regions some distant apart of the *TNFSF13B* gene are influencing its expression.

In conclusion, in the present study we have failed to identify an association between the *TNFSF13B* functional variant previously associated with autoimmunity and two immune-related diseases, GCA and SSc. Thus, this genetic variant does not seem to be responsible for the increased levels of BAFF found in these disorders.

## Supporting information

S1 Table Results of the meta-analysis of the different GCA cohorts after stratification of patients according to their main clinical characteristics. OR, odds ratio. PMR, polymyalgia rheumatica; JC, jaw claudication; VM, visual manifestations; OR, odds ratio. ^a^OR for the minor allele.
(PDF)

S2 Table Results of the meta-analysis of the different SSc cohorts after stratification of patients according to their main clinical characteristics. lSSc, limited cutaneous SSc; dSSc, diffuse cutaneous SSc; ACA, anticentromere antibodies; ATA, antitopoisomerase antibodies OR, odds ratio. ^a^OR for the minor allele.
(PDF)
