# Supplementary material for: A TNFSF13B functional variant is not involved in systemic sclerosis and giant cell arteritis susceptibility
Source: PLoS One. 2018 Dec 26;13(12):e0209343. doi: 10.1371/journal.pone.0209343 (PMC6306228; doi:10.1371/journal.pone.0209343)
Supplement: S1 Table — OR, odds ratio. PMR, polymyalgia rheumatica; JC, jaw claudication; VM, visual manifestations; OR, odds ratio. aOR for the minor allele. (PDF) [file pone.0209343.s001.pdf]

S1 Table. Results of the meta-analysis of the different GCA cohorts after stratification of patients according to their main clinical characteristics.

	Meta-analysis			
**Subgroup (N)**	***P*-value**	**OR [CI 95%][a]**	**Q test (p-value)**	**I^2**
Controls (n=5,160)	-	-	-	-
PMR+ (n=638)	0.241	0.82	0.194	36%
PMR- (n=974)	0.738	0.96	0.770	0%
JC+ (n=758)	0.250	0.84	0.975	0%
JC- (n=865)	0.661	0.94	0.157	43%
VM+ (n=566)	0.430	0.87	0.444	0%
VM- (n=950)	0.210	0.85	0.826	0%

PMR, polymyalgia rheumatica; JC, jaw claudication; VM, visual manifestations; OR, odds ratio.

[a] OR for the minor allele.
