# Supplementary material for: A TNFSF13B functional variant is not involved in systemic sclerosis and giant cell arteritis susceptibility
Source: PLoS One. 2018 Dec 26;13(12):e0209343. doi: 10.1371/journal.pone.0209343 (PMC6306228; doi:10.1371/journal.pone.0209343)
Supplement: S2 Table — lSSc, limited cutaneous SSc; dSSc, diffuse cutaneous SSc; ACA, anticentromere antibodies; ATA, antitopoisomerase antibodies OR, odds ratio. aOR for the minor allele. (PDF) [file pone.0209343.s002.pdf]

S2 Table. Results of the meta-analysis of the different SSc cohorts after stratification of patients according to their main clinical characteristics.

	Meta-analysis			
**Subgroup (N)**	***P*-value**	**OR [CI 95%][a]**	**Q test (p-value)**	**I^2**
Controls (n=4,690)	-	-	-	-
lSSc (n=2,692)	0.440	1.07	0.907	0%
dSSc (n=971)	0.909	0.99	0.536	0%
ACA+ (n=1,758)	0.431	1.08	0.302	17%
ACA- (n=2,120)	0.401	1.08	0.995	0%
ATA+ (n=909)	0.634	1.06	0.884	0%
ATA- (n=2,917)	0.462	1.07	0.391	0%

lSSc, limited cutaneous SSc; dSSc, diffuse cutaneous SSc; ACA, anticentromere antibodies; ATA, antitopoisomerase antibodies OR, odds ratio.

[a] OR for the minor allele.
